# Physico-Chemical Condition Optimization during Biosynthesis lead to development of Improved and Catalytically Efficient Gold Nano Particles

**DOI:** 10.1038/srep27575

**Published:** 2016-06-08

**Authors:** Madhuree Kumari, Aradhana Mishra, Shipra Pandey, Satyendra Pratap Singh, Vasvi Chaudhry, Mohana Krishna Reddy Mudiam, Shatrunajay Shukla, Poonam Kakkar, Chandra Shekhar Nautiyal

**Affiliations:** 1 CSIR-National Botanical Research Institute, Rana Pratap Marg, Lucknow, 226 001, India; 2 CSIR-Indian Institute of Toxicology Research, Vishvigyan Bhawan 31, Mahatma Gandhi Marg, Lucknow, 226 001, India

## Abstract

Biosynthesis of nanoparticles has gained great attention in making the process cost-effective and eco-friendly, but there are limited reports which describe the interdependency of physical parameters for tailoring the dimension and geometry of nanoparticles during biological synthesis. In the present study, gold nanoparticles (GNPs) of various shapes and sizes were obtained by modulating different physical parameters using *Trichoderma viride* filtrate. The particles were characterized on the basis of visual observation, dynamic light scattering, UV-visible spectroscopy, transmission electron microscopy, fourier transform infrared spectroscopy, and X ray diffraction. While the size varied from 2–500 nm, the shapes obtained were nanospheres, nanotriangles, nanopentagons, nanohexagons, and nanosheets. Changing the parameters such as pH, temperature, time, substrate, and culture filtrate concentration influenced the size and geometry of nanoparticles. Catalytic activity of the biosynthesized GNP was evaluated by UV-visible spectroscopy and confirmed by gas chromatography-mass spectrometric analysis for the conversion of 4-nitrophenol into 4-aminophenol which was strongly influenced by their structure and dimension. Common practices for biodegradation are traditional, expensive, require large amount of raw material, and time taking. Controlling shapes and sizes of nanoparticles could revolutionize the process of biodegradation that can remove all the hurdles in current scenario.

In the rapidly growing area of nanomaterial research, gold nanoparticles have entered into a new arena, opening new possibilities in catalysis^1^, diagnostics and biomedicine^2^, optics^3^,^12^, imaging^4^, electronics^13^, sensing^5^,^6^, and agriculture^7^. The functions of gold nanoparticles heavily rely upon their structure, size and morphology, so finding a way to modulate nanoparticles biosynthesis is an important aspect of the research^8^,^9^,^10^. Shape and symmetry-dependent mechanical properties of metallic gold on the nanoscale has already been elucidated by Mahmoud *et al.*^11^.

Despite tremendous progress in the synthesis of gold nanocrystals of various shapes and sizes with good yield and monodispersity *via* chemical routes^14^,^15^, the conventional approaches remain unimplementable due to requirement of harsh chemicals, high temperature, pressure and are also non eco-friendly^16^,^17^. Biogenic synthesis of nanoparticles has emerged as an absolute alternative and green approach, making the process cheaper and safer^15^. Several reports are available for biosynthesis of gold nanoparticles by plants, fungi, bacteria, and actinomycetes. Fungi like *Verticillium* sp., *Phoma* sp., *Fusarium oxysporum, Aspergillus fumigatus,* and *Rhizopus oryzae* are considered to be the best source for synthesis of nanoparticles^15^. They are the producers of significant amounts of proteins and secondary metabolites secreted extracellularly, which act as both reducing and stabilizing agent for nanoparticles biosynthesis^16^. Their requirements for growth are simple, easy to manipulate and downstream processing is much easier as compared with other microorganisms. In a previous study carried out in our group, *Trichoderma viride* and *Hypocrea lixii* have been reported for synthesis of gold nanoparticles within 10 min^16^.

Despite significant progress in biosynthesis of nanoparticles, little has been done on controlling the shape of the metal nanoparticles by biological routes. Gold nanoparticles of various shapes and sizes have been synthesized by *Piper betle* and alfalfa extracts^18^,^19^. Some of the reports describe the effect of physical parameters on formation of vivid range of GNPs^1^,^20^,^21^ but the combinatorial effect of biological and physical parameters affecting shape and size of nanocrystals have not been yet elucidated.

Each physical and biological parameter plays a different role while governing the rules for dimension and geometry of nanoparticles. Cell free extract being source of reducing and stabilizing agent can affect the nucleation and growth of nanoparticles. High concentration of cell free extract can enhance nucleation at a high rate because of the high concentration of protein^20^. Similarly, high concentration of gold ion, nanoparticles formed are thermodynamically unstable because of insufficient ligand protection^15^,^20^, making it very necessary to obtain the right balance between cell free extract and gold ion concentration. The oxidation/reduction state of proteins and enzymes present in cell free extract are highly dependent upon the pH making it a substantial factor to determine the shape and size of the nanoparticles. Along with cell free extract, gold ion concentration and pH, reaction time and temperature can also affect the rate of nucleation and growth of nanoparticles. As the reaction temperature increases, the reaction rate increases consuming gold ions to form the nuclei, thereby enhancing the biosynthesis process^18^. Varying reaction time can lead to variation in growth rate of seed particle generating multi-shaped nanoparticles^8^. It is very clear that a single parameter cannot decide the fate of nuclei; rather it is the balance between all the parameters which can generate different shapes and sizes of nanoparticles.

The urgent need of the future is to develop an eco-friendly approach for highly selective reaction systems using heterogeneous catalysts^22^. The catalytic properties of gold nanoparticles are highly dependent upon its morphology^23^ but the precise conditions for maximum activity and selectivity of nanocatalyst remains doubtful^24^. In the present study, to the best of our knowledge, interdependent role of pH, time, temperature, concentration of cell free extract, and gold salt on generating different shapes and sizes of gold nanoparticles is reported for the first time by using cell-free extract of *T. viride* (MTCC 5661) both, as a reducing and capping agent. An attempt has been made to obtain unique balance of physical parameters to set a strategy for selection of specific structures of gold nanoparticles for enhancing catalytic properties in degradation of organic pollutants.

## Results and Discussion

### Biosynthesis and characterization of gold nanoparticles

**Figure 1:**
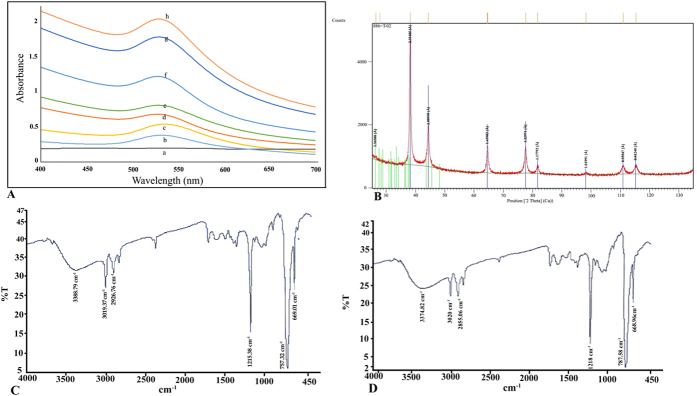
(**A**) UV-vis spectrum of biosynthesized gold nanoparticles at time intervals of (a) 0 min (b) 1 min (c) 2 min (d) 5 min (e) 10 min (f) 24 h (g) 48 h (h) 72 h (**B**) XRD pattern of biosynthesized gold nanoparticles (**C**) FTIR spectra of cell free extract alone and (**D**) gold nanoparticles biosynthesized by cell free extract of *Trichoderma viride*.

Biosynthesis of gold nanoparticles was evident from the color change of the reaction mixture and confirmation was done by UV-visible spectroscopy (Fig. 1A). The reduction process was very quick and color change was evident 10 min after addition of HAuCl_4_^16^. Gold nanoparticles are known to exhibit a range of colors according to their shapes and sizes, due to surface plasmon resonance and accordingly can be characterized by obtaining the characteristic peak in 500–600 nm range^25^. The crystalline nature of gold nanoparticles was confirmed by X-ray diffraction (XRD) studies (Fig. 1B). The XRD pattern recorded from the solution cast film of *T. viride* reduced gold nanoparticles, showed a very intense Bragg reflection at 38.6, 44.38, 64.57 and 77.5 degree corresponding to (111), (200), (220) and (311) crystal lattice of face-centered cubic (FCC) gold. Fourier-transform infrared spectroscopy (FTIR) was carried out to identify the biological moieties involved in biosynthesis. The FTIR spectrum of the cell-free extract and the biosynthesized gold nanoparticles (Fig. 1C,D) exhibited intense and distinct absorption bands at 757.58 and 1215.2 cm^−1^. The intense absorbance at 757.58 cm^−1^ corresponds to C–H “oop” of aromatics while 1215 cm^−1^ can be assigned to C–N stretch of aliphatic amines. The FTIR spectrum also showed bands at 668.96, 2855.06, 3020, and 3374.82 cm^−1^ with some other bands. The band at 668.962 cm^−1^ corresponds to C–Br stretch of alkyl halides and 2855.06 cm^−1^ demonstrates H–C=O: C–H stretch of aldehydes. The medium band at 3020 cm^−1^ demonstrates =C–H stretch of alkenes and band at 3374.82 cm^−1^can be assigned to O–H stretch of alcohols and phenols. The results clearly indicated the role of several biological moieties, including proteins and organic compounds which might be the major components of secondary metabolites in biosynthesis of nanoparticles. Secondary metabolites of *Trichoderma* are rich in non-ribosomal peptides, peptaibols, siderophores, polyketides, terpenoids/steroids, pyrones and hydrolytic enzymes^25^. These organic moieties contain several phenolic groups and their derivatives which can take part in redox reaction generating quinones and donating electrons. These electrons can further reduce and stabilize the metallic ions into nanoparticles. Many reports of biosynthesis have demonstrated the role of organic compounds and proteins in reduction and stabilization of nanostructures^15^,^25^.

### Morphological observations of biosynthesized gold nanoparticles

A wide range of colors were observed at different conditions of pH, temperature, reaction time, salt, and extract concentrations (Fig. S1). Dynamic light scattering (DLS) size also indicated the reduction of gold ions and their conversion into nanoparticles of different sizes with good monodispersity (Figs S2 and S3). DLS provides the size of whole conjugate including size of individual nanoparticles and the ligand shell surrounding them, thus providing larger size than obtained during TEM measurements^15^. Effect of each parameter with respect to the other was studied in detail (Table 1) and a mechanisms/suitable conditions were proposed for obtaining monodispersed population of a single geometry.

### Effect of concentration of cell free extract

**Figure 2:**
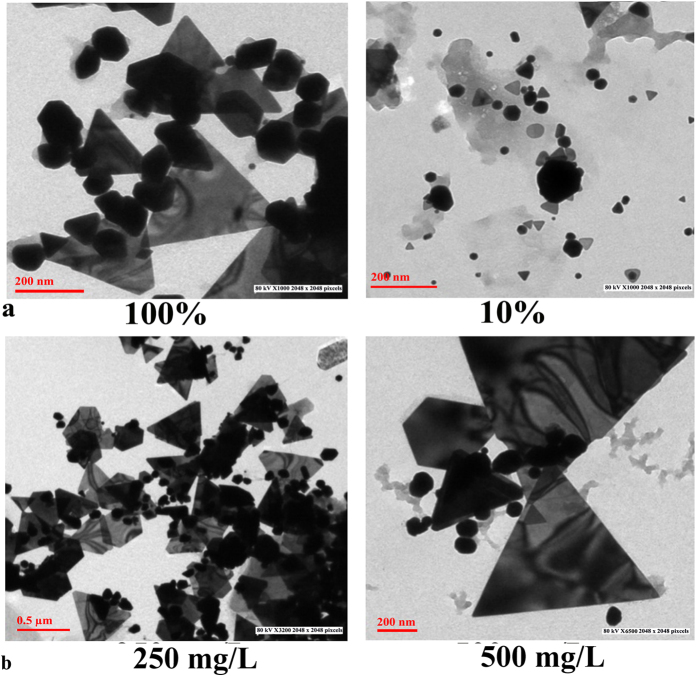
TEM micrographs of (**a**) G100.250.7.72.30 and G10.250.7.72.30 showing effect of different concentrations of cell free extracts (100 and 10%) (**b**) G10.250.7.72.40 and G10.500.7.72.40 showing effect of different concentrations of HAuCl_4_ (250 and 500 mg/L) on shapes and sizes of gold nanoparticles.

Among the three concentrations of cell free extract chosen viz. 100, 50, and 10%, (sample code G10.250.7.72.30, G50.250.7.72.30 and G100.250.7.72.30) all were able to form nanoparticles with 250 mg/L of HAuCl_4_ (Figs 2a, S4a and S10). With decreasing cell free extract concentration, the size of the particles decreased accordingly, though there was no significant difference in the shape of the particles synthesized. Direct application of cell free extract without any dilution (G100.250.7.72.30) yielded particles of the size 197.58 ± 68 nm, while particles formed by 50% of cell free extract (G50.250.7.72.30) had a size of 104 ± 55 nm. The smallest size (34 ± 20 nm) was recorded with 10% extract (G10.250.7.72.30) (Figs 2a and S10). Earlier reports of Das *et al.*^20^ and Song *et al.*^26^ have suggested the reduction in particle size with increase in cell free extract concentration. Contrary to these results, in this study by decreasing the concentration of reducing agent (cell free extracts), reduction in particle size was obtained. Higher concentration of reducing agents might increase the catalytic activity which leads to faster reaction, thus size of nanoparticles increased in presence of higher concentration of cell free extract. Higher concentration of cell free extract increased the concentration of reducing agent, thus increasing the reaction rate which resulted in rapid growth of nanoparticles^27^, so increase in size was observed as the concentration increased.

### Effect of concentration of HAuCl_4_

Two concentrations of gold salt viz. 250 and 500 mg/L were taken and their effect on shapes and sizes at different temperatures viz. 30, 40 and 50 °C were observed with 10% cell free extract. At the lower concentration, particles formed were smaller as compared to the particles formed at 500 mg/L (Figs 2b, S4b and S11). Temperature also played a crucial role at both the concentrations. At 30 °C, 250 mg/L, (G10.250.7.72.30) mixed population of particle were synthesized having mean size of 34 nm, while at 500 mg/L (G10.500.7.72.30), particles were predominantly hexagons of 85.2 nm. At 40 °C, nanoparticles were triangular at both concentrations, while the mean size increased from 164 (G10.250.7.72.40) to 335 nm (G10.500.7.72.40) when concentration of gold salt was increased from 250–500 mg/L. At 50 °C, very large triangles of mean size 699 nm were formed at 500 mg/L (G10.500.7.72.50), while triangles of 273.6 nm were biosynthesized at 250 mg/L (G10.250.7.72.50). At higher concentration of gold salt, the cell free extract concentration becomes less, resulting in insufficient capping and stabilizing action of reducing agents^20^. This situation resulted in exposure of different facets on surface, which after random collisions and fusion of nuclei formed larger nanoparticles. As the temperature was increased, there was an increase in the rate of reaction resulting in the formation of small nuclei quickly^26^, which might further collide rapidly and randomly to form larger particles at higher concentrations and higher temperature at a given interval of time.

### Effect of pH

**Figure 3:**
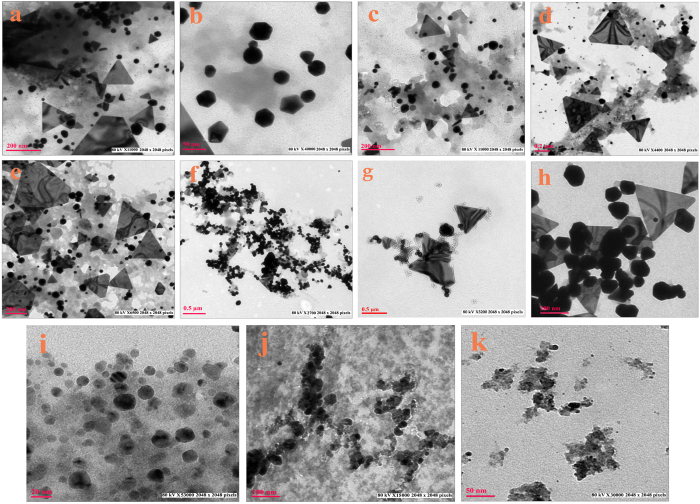
TEM micrographs of (**a**) G10.250.5.72.30 (**b**) G10.250.5.5.72.30 (**c**) G10.250.6.72.30 (**d**) G10.250.6.5.72.30 (**e**) G10.250.7.72.30 (**f**) G10.250.7.5.72.30 (**g**) G10.250.8.72.30 (**h**) G10.250.8.5.72.30 (**i**) G10.250.9.72.30 (**j**) G10.250.9.5.72.30 (**k**) G10.250.10.72.30 showing effect of different pH on shapes and sizes of gold nanoparticles (See Table 1 for detail).

Among the growth parameters, pH plays a substantial role on the oxidation state and reducing power of enzymes and secondary metabolites present in cell free extract^20^. A wide pH range was tested for biosynthesis of gold nanoparticles at 30 °C, time interval of 72 h and HAuCl_4_ concentration of 250 mg/L (Figs 3 and S12). At pH 5.0 (G10.250.5.72.30) particles of varying shapes i.e large prisms, triangles, pentagons, hexagons and rods were synthesized, with size range of 5–200 nm. As the pH was increased, the shape of the particles changed to star/hexagonal at pH 5.5 [(G10.250.5.5.72.30) (10–100 nm)], to triangles at pH 6.0–8.0[(G10.250.6.72.30, G10.250.6.5.72.30, G10.250.7.72.30, G10.250.7.5.72.30 and G10.250.8.72.30) (5–200 nm)], which were further converted into penta/hexagons of size 50–80 nm at pH 8.5(G10.250.5.72.30). When the pH was further increased to 9.0 (G10.250.9.72.30), particle size went down to 3–10 nm and shape changed to spherical and it changed to irregular at pH 9.5 and 10.0(G10.250.9.5.72.30 and G10.250.10.72.30). It was observed that the particle size decreased with increase in pH. At lower pH, a mixed population was synthesized, similar to the observations of Armendariz *et al.* and Sneha *et al.*^18^,^28^ who demonstrated the synthesis of tetrahedral, hexagonal, decahedral and rod-shaped nanoparticles at pH 2.0–6.0. Each methodology involves two steps for gold nanoparticles synthesis: (I) nucleation and (II) crystal growth^27^,^29^. At lower pH, repulsion between negatively charged AuCl_4_^−^ ions and carboxylic group of extract is reduced resulting in uncontrolled nucleation of seeds and formation of larger mixed shape particles^18^. Lower pH, with due course of time allows coagulation of smaller nuclei resulting in larger colloids^30^. After 72 h of reaction time, at pH 5, smaller nuclei aggregated, forming larger particles. At neutral range of pH (6.0–8.0), 70–80% of population obtained consisted of nanotriangles and prisms. This range of pH is predominated by AuCl_4_^−^30 and most of the active biological moieties, predominantly synthesized nanoprism in due course of time. As the pH was increased to 9.0, ≥ 90% of the population consisted of spheres with significant decrease in size to 3–10 nm. Earlier, role of higher pH in synthesis of small nanospheres has been discussed by many researchers^18^,^20^,^31^,^32^.

At higher pH, Cl^−^ ions present in AuCl_4_^−^ get substituted by OH^−^ present in cell free extract resulting in repulsion between the two negatively charged moieties of cell free extract and gold ions, thereby reducing the possibility of further growth of nuclei maintaining them as small spheres.

### Effect of reaction time

**Figure 4:**
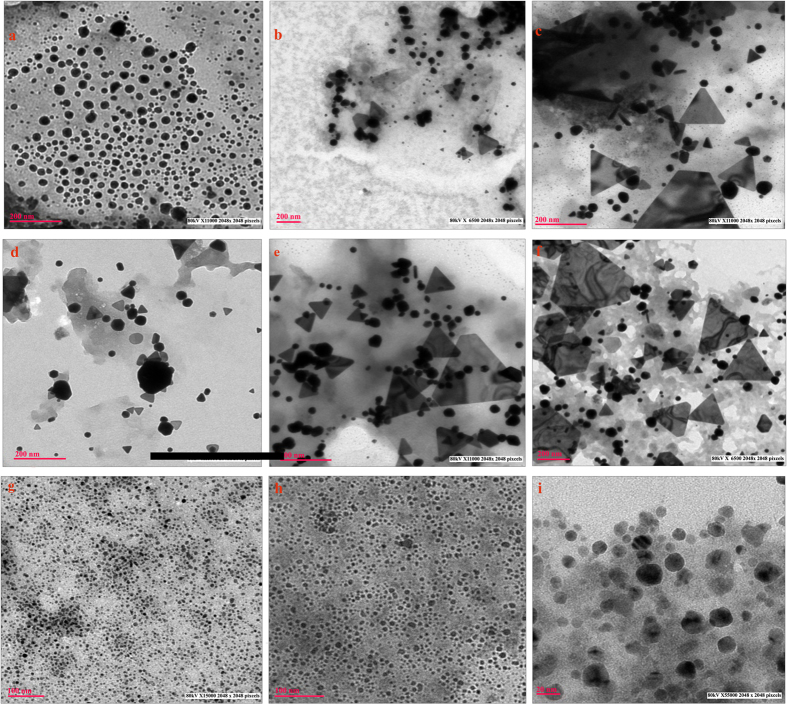
TEM micrographs of (**a**) G10.250.5.24.30 (**b**) G10.250.5.48.30 (**c**) G10.250.5.72.30 (**d**) G10.250.7.24.30 (**e**) G10.250.7.48.30 (**f**) G10.250.7.72.30 (**g**) G10.250.9.24.30 (**h**) G10.250.9.48.30 (**i**) G10.250.9.72.30 showing effect of different time intervals at pH on shapes and sizes of gold nanoparticles (See Table 1 for detail).

Effect of time on morphology of gold nanoparticles biosynthesized was studied at 24, 48 and 72 h of time interval at three different pH 5.0, 7.0 and 9.0 (Figs 4 and S13, Table 1). After 24 h, at pH 5.0 (G10.250.5.24.30), 100% of the particles synthesized were small spheres (7–24 nm), indicating the initiation of nucleation, which was gradually followed by synthesis of mixed population of spheres, triangles and prisms of larger size (7–120 nm) after 48 h (G10.250.5.48.30) due to growth of crystals. After 72 h, (G10.250.5.72.30) particles formed were predominantly triangles and prisms of 20–400 nm size. Similarly, at pH 7.0, after 24 h, (G10.250.7.24.30) the particles were smaller (5–60 nm) with mixed population of triangles and spheres, and thereafter there was formation of nanoprism after 48 h (G10.250.7.48.30) and 72 h(G10.250.7.72.30) of size 200 nm. Mukherjee *et al.*^21^ demonstrated in their study the evolution of morphology of gold nanoparticles from nano-spheres to triangular nanoprisms with increase in time in *T. asperellum*. During the period of crystal growth, spheres might have fused to form triangles whose further fusion yielded large nanoprisms. The time factor was negligible for synthesis of different shapes and sizes of nanoparticles at pH 9. Shapes and sizes were unaffected at higher pH for long duration (G10.250.9.24.30, G10.250.9.48.30 and G10.250.9.72.30), because of repulsion between dominant negatively charged groups of cell free extract and negatively charged AuCl_4_^−^ ions resulting in no change in geometry and dimension of nanoparticles biosynthesized even after 72 h of reaction.

### Effect of temperature

**Figure 5:**
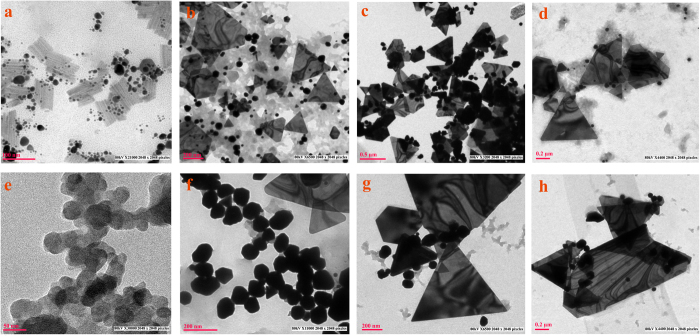
TEM micrographs of (**a**) G10.250.7.72.20 (**b**) G10.250.7.72.30 (**c**) G10.250.7.72.40 (**d**) G10.250.7.72.50 (**e**) G10.500.7.72.20 (**f**) G10.500.7.72.30 (**g**) G10.500.7.72.40 (**h**) G10.500.7.72.50 showing effect of different reaction temperatures at pH 7.0, at different concentrations of HAuCl_4_ on shape and size of gold nanoparticles (See Table 1 for detail).

To study the effect of temperature on morphology of gold nanoparticles, experiments were carried out at 20, 30, 40 and 50 °C at different gold ion concentrations (250 and 500 mg/L) and pH (5.0, 7.0 and 9.0) (Figs 5 and S5, S6, S14, S15, S16; Table 1).

**Figure 6:**
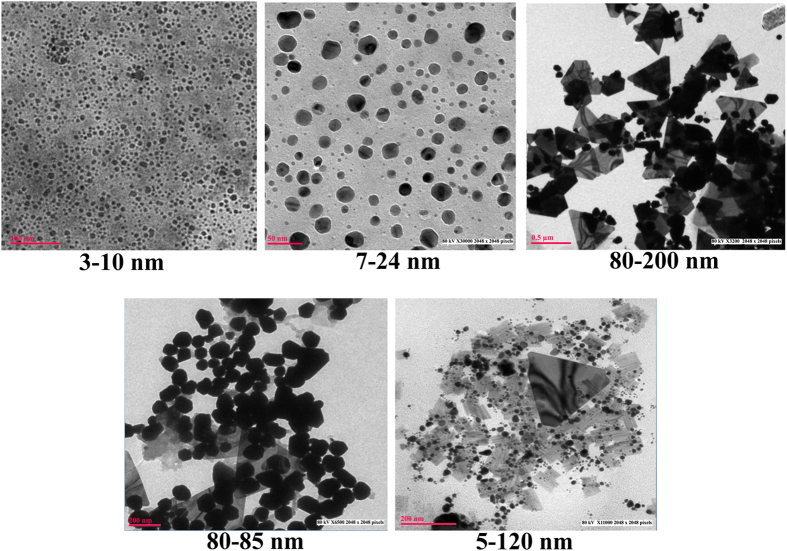
TEM micrographs of biosynthesized gold nanoparticles of different morphologies (**a**) G10.250.9.24.30 (spherical,3–10 nm) (**b**) G10.250.5.24.30 (spherical,7–24 nm) (**c**) G10.250.7.72.40, (triangles and prisms,80–200) (d) G10.500.9.72.40, (penta and hexagonal,80–85 nm) (**e**) G10.250.7.72.20, (sheets,5–120 nm).

Biosynthesis of gold nanoparticles at 50 °C yielded large prisms of 200–600 nm irrespective of the pH and gold ion concentrations. This temperature was neither low nor very high for growth of nuclei. It was an optimum temperature for generation of activation energy required for catalytic activity to synthesize larger prisms. At 40 °C, and at pH 7.0 (G10.250.7.72.40) and 5.0 (G10.250.5.72.40), there was formation of mixed population, while percentage of nanoprisms was more in 500 mg/L (G10.500.7.72.40 and G10.500.5.72.40) of HAuCl_4_ concentration (Figs 5, S5 and S14). However, at pH 9.0 (G10.500.9.72.40), 82% population observed was pentagons and hexagons. A transition phase was created during nucleation and crystal growth at 40 °C and pH 9.0. This situation becomes favorable for formation of pentagons and hexagons (Fig. 6). Crystal growth without any hindrance could lead to formation of nanoprisms at lower pH, but at higher pH, due to decrease in crystal growth because of repulsion between the negatively charged groups, evolution in morphology was slow, resulting in penta/ hexagonal particle synthesis.

Similarly, rate of reaction and nucleation was slow at 30 °C, pH 9.0 (G10.250.9.72.30). Both the reaction parameters were favorable for the synthesis of small spherical nuclei of 2–30 nm. At pH 5.0 (G10.250.5.72.30) and 7.0 (G10.250.7.72.30), mixed population of triangle, spheres, rods and penta/hexagons were obtained which might be a result of evolution of spherical seeds which were obtained earlier at 24 h at similar reaction conditions. At 500 mg/L of HAuCl_4_ concentration, particles were predominantly pseudospheres at pH 5.0 (G10.500.5.72.30) and penta/hexagons at pH 7.0 (G10.500.7.72.30).

When the reaction was carried out at 20 °C and at the pH 9.0 (G10.250.9.72.20), pH factor played the dominant role for the formation of small nanoparticles (10–60 nm). At pH 7.0 (G10.250.7.72.20), the geometry of the particles became more uniform. 70% of the total population consisted of nanosheets, not reported yet by any biological methods, while the rest comprised of pseudospheres of 10–50 nm. At pH 5.0 (G10.250.5.72.20), nanoparticles biosynthesized consisted of a mixed population of triangles, prisms, rods and penta/hexagons of various sizes at both the HAuCl_4_ concentrations. The mechanism involved behind this phenomenon is yet to be understood.

### Conditions for synthesis of multishaped gold nanoparticles and the proposed mechanism

**Figure 7:**
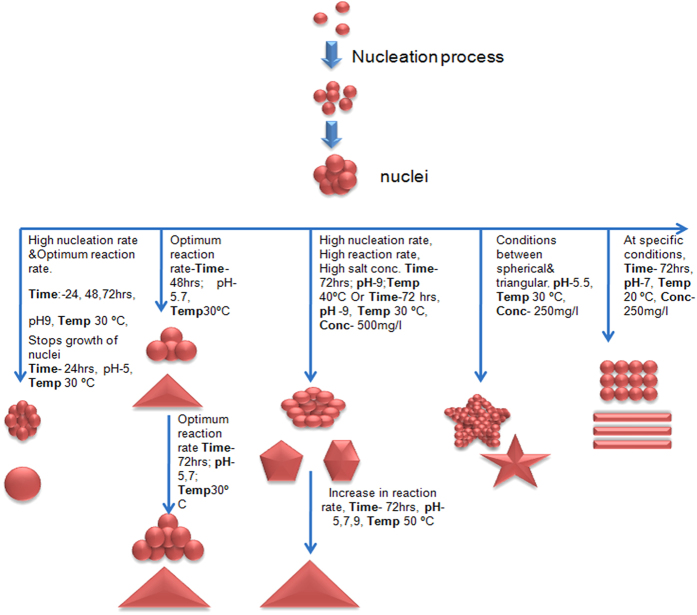
Schematic diagram representing mechanism of formation of gold nanoparticles of different shapes and sizes under different reaction conditions.

Suitable conditions were deciphered to synthesize the desired shape and size of nanoparticles by regulating combination of pH, reaction time, temperature, concentration of gold salts, and cell free extract (Table 1 and Fig. 7).

#### Spherical nanoparticles

G10.250.9.24.30, G10.250.9.48.30, G10.250.9.72.30 and G10.250.5.24.30 were the 100% homogeneous spherical GNPs (Fig. S7). For obtaining monodispersed population of nanospheres, it is necessary to stop the growth of nuclei. This can either be achieved at very high temperature^16^, or at high pH at 30 °C as reported in our study, where repulsion was higher between the negatively charged biological moieties and chloroaurate ions. Spherical nanoparticles can also be formed by inhibiting further crystal growth after development of nuclei by stopping reaction at an early stage (Fig. S7).

#### Triangular nanoparticles and nanoprisms

G10.250.7.48.30, G10.250.5.72.40 and G10.250.7.72.40 resulted in formation of nanotriangles and nanoprisms (Fig. S8). At pH 5.0 and 7.0, there was no hindrance in the growth of crystals at 40 °C, 72 h, leading towards formation of nanotriangles. After 48 h, nanotriangles were observed which were smaller in size. When the incubation was beyond 24 h, the nuclei increased in size (upto 400 nm) but shape remained the same. Mukherjee *et al.*^21^ have also demonstrated the evolution of morphology from sphere to triangles in a time dependent manner. Time plays a major role in nanotriangle formation but the major constraint associated with this is the growth of the crystal along with formation of nanoprisms. Biosynthesis of smaller triangles and prisms below 50 nm still remains a mammoth task to be achieved.

#### Penta and hexagon nanoparticles

Favourable situations for biosynthesis of penta and hexagons were obtained at G 10.500. 9.72.40 (80–85 nm) and at G10.500.7.72.30 (Fig. S8). In the first case, at higher pH, growth of crystal is retarded while the rate of reaction is high at high temperature, so after 72 h of reaction, spherical nuclei could have been converted into hexagons and pentagons instead of triangles, because of two opposing forces of temperature and pH were working in synergism. In the second case, at pH 7.0 and 30 °C, all conditions were favorable for the reaction where concentration of gold salt might play an important role. Insufficient amount of the cell free extract to cover all gold ions led to incomplete capping^23^ of salt, leading to selective growth for the construction of penta and hexagonal nanoparticles. Star shaped hexagons were observed at pH 5.5 indicating the evolution of morphology from spherical seeds to triangles.

#### Nanosheets

Nanosheets were synthesized at pH 7.0, 250 mg/L concentration of gold salt, 20 °C after 72 h of reaction (G10.250.7.72.20) (Fig. S8). However, on keeping the conditions same, when temperature was changed to 30 and 40 °C, nanotriangles were formed. There might be fusion of small nuclei to form straight chains, which when clubbed together gave the appearance of a sheet. There might also be the effect of temperature on formation of nanosheets, the mechanism of which still needs to be explored.

### Effect of different morphologies of GNP in catalytic conversion of 4-nitrophenol

**Figure 8:**
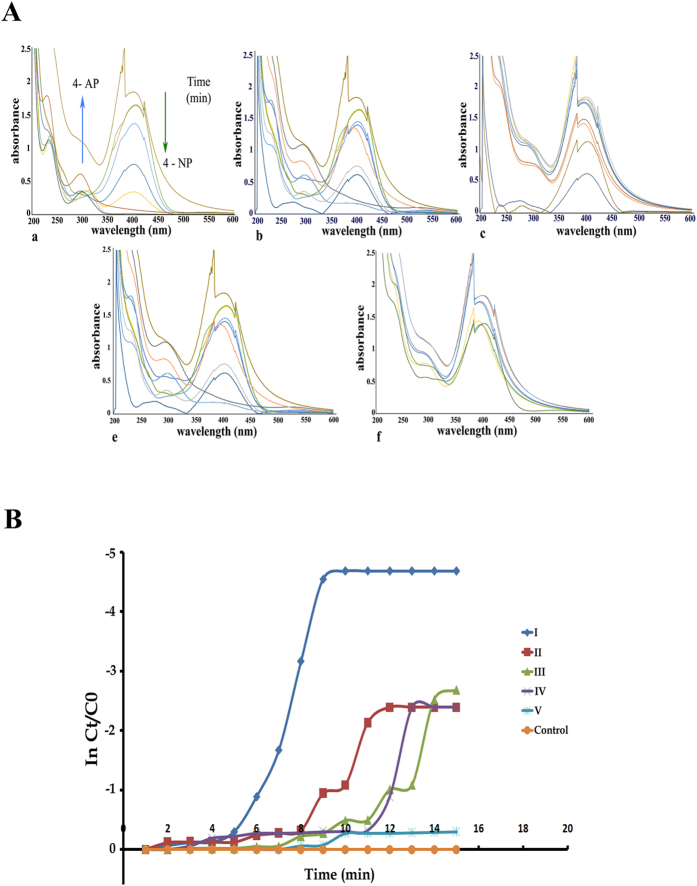
**(A)**Time dependent UV-vis absorption spectra for the reduction of 4-NP by NaBH_4_ to 4-AP for a time period of 5 min in the presence of biosynthesized gold nanoparticles of different shapes and sizes (a) G10.250.9.24.30 (b) G10.250.5.24.30 (c) G10.250.7.72.40 (d) G10.500.9.72.40 (e) G10.250.7.72.20; (**B).** Plots of ln(C_t_/C_0_) vs Time for the reduction of 4-NP by NaBH_4_ to 4- AP in the presence of biosynthesized gold nanoparticles of different shapes and sizes (a) G10.250.9.24.30 (b) G10.250.5.24.30 (c) G10.250.7.72.40 (d) G10.500.9.72.40 (e) G10.250.7.72.20 (f) control without addition of gold nanoparticles.

Biogenic nanoparticles have significantly higher catalytic activity due to presence of protein corona on their surface which acts as an effective host for the substrate^33^. Different shapes of particles obtained in our study viz: spheres, triangles, penta/hexagons and sheets (Fig. 6) were evaluated for their catalytic activity. Among the particles of different shapes, spherical gold nanoparticles (3–10 nm) acted as the best catalyst, demonstrated a sharp decrease in absorbance at 400 nm followed by penta/hexagonal, triangles and nanosheets (Fig. 8A). The decrease in nitrophenolate ion and disappearance of yellow colour indicated that 4- nitrophenol (4-NP) was converted to 4-aminophenol (4-AP) within 5 min, just after addition of spherical GNPs to the reaction mixture, while it took 30 min and 120 min for complete degradation of 4-NP in case of penta/hexagonal, and triangular nanoparticles respectively (Fig. 8B).

4-NP exhibits an absorption peak at 400 nm in alkaline condition because of formation of nitrophenolate ion^34^. In the presence of gold nanoparticles as biocatalyst, 4-NP was converted into 4-AP resulting in decrease in absorbance at 400 nm and increase in 300 nm due to formation of 4-AP^15^.

Since the concentration of NaBH_4_ is much higher than 4-NP, the reduction rate can be assumed to be independent of NaBH_4_ concentration. Therefore, the catalytic rate constant (K) in this case can be evaluated by studying the pseudo-first-order kinetics with respect to 4-NP concentration. The kinetics study demonstrated that the rate constant was maximum in spheres (3.94 × 10^−3^ section^−1^), followed by penta/hexagonal particles (2.4 × 10^−3^ section^−1^), triangles (2.3 × 10^−3^ section^−1^), and nanosheets (0.79 × 10^−3^ section^−1^). The higher catalytic activity of nanospheres may be attributed to their smaller size and large volume to surface area ratio in comparison to nanoparticles of other shapes, providing maximum number of reaction sites for catalysis. The smaller size results in higher number of active sites with high surface-to-volume ratios^35^, while different shapes expose different facets having different selectivity and reactivity^36^. Tetrahedral nanoparticles, having sharp edges and corners, composed entirely of (111) facets are considered as most reactive, while cubic nanoparticles, and composed entirely of (100) facets with fewer edges and corners are less reactive. Spherical nanoparticles are a mixture of both (111) and (100) facets and have corners and edges at the interfaces of these facets possess the intermediate position in reactivity^37^. Spherical nanoparticles of 7–24 nm demonstrated highest rate of reaction because of their small size and intermediate selectivity while penta and hexagonal nanoparticles, demonstrated good catalytic activity, because of their reactive facets and sharp corners and edges, despite their larger size (80–85 nm) as compared to spherical particles.

Further, when spherical gold nanoparticles of two different sizes were compared, it was found that particles of 3–10 nm (6.75 × 10^−3^ section^−1^), had higher rate constant than particles of 7–24 nm (3.94 × 10^−3^ section^−1^).

### Comparison of different sizes of spherical GNP for catalytic activity by GC-MS/MS analysis

**Figure 9:**
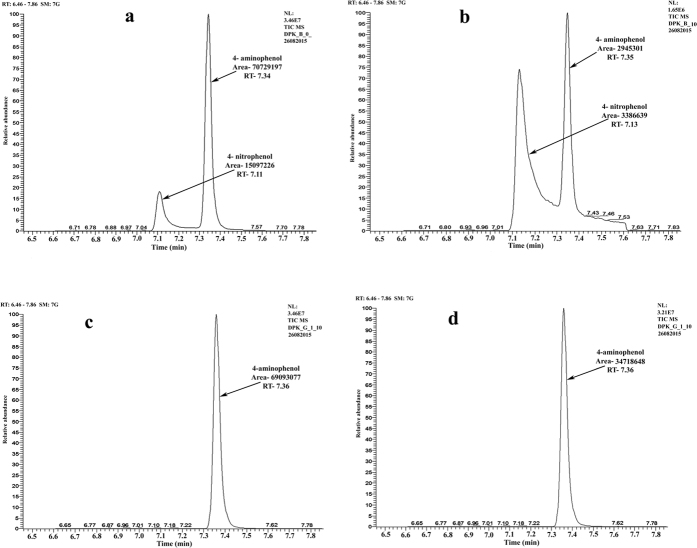
Catalytic activity of different spherical sizes of GNPs in presence of reducing agent NaBH_4_ (**a**) Control at 0 min showing peak of 4-NP {Retention time 7.11 and the area 15097226} and peak of 4-AP {RT- 7.34, area- 70729197} (**b**) Control at 10 min showing the presence of peak of 4-NP {RT- 7.13, area- 3386639}and 4-AP {RT- 7.35, area- 2945301} (**c**) G1(3–10 nm) showing final conversion of 4-NP into 4-AP at 10 min {RT- 7.36, area-69093077} (**d**) G2 (7–24 nm) showing only 4-AP {RT- 7.36, area-34718648} at 10 min.

The catalytic activtity of two spherical nanoparticles showing maximum rate constant (3–10 and 7–24 nm) were further evaluated by gas chromatography-mass spectrometric analysis (GC-MS). Mixture of 4-NP and 4-AP; obtained at retention time 7.13 and 7.34 respectively (Fig. 9). In blank transformation of 4-NP (RT at 7.13) to 4-AP (RT at 7.34) was also observed without addition of gold nanoparticles after 5 min, but it was not stable and we further observed the starting material (4-NP, RT-7.14) at 10 min along with the product (4-NP, RT- 7.34). This may be due the instability of the product in the absence of any catalyst (Fig. S9). While in treatment of both spherical GNP (3–10 and 7–24 nm) GC-MS/MS results confirmed the complete conversion of 4-NP into 4-AP with appearance of single metabolite of 4-AP at RT 7.36 and disappreance of 4-NP after 5 min of addition of spherical gold nanoparticles. This was clearly evident from the complete conversion of 4-NP into 4-AP, when we use spherical nanoparticles as catalyst which is proved by GC-MS analysis of the reaction mixture. In agreement with the earlier results, smaller nanoparticles (3–10 nm) acted as better catalyst depicting almost double the peak area of 4-AP than the larger spherical nanoparticles (7–24 nm) after 5 and 10 min of addition of GNPs to the reaction mixture (Fig. S9). Though spherical particles of both sizes were excellent catalyst, smaller nanoparticles (3–10 nm) emerged as a better catalytic agent for conversion of 4-NP into an eco-friendly product (4-AP) and it helps to degrade the toxic organic pollutants.

Particles below 10 nm have previously been known for their excellent catalytic properties^24^,^38^. Decrease in size below 10 nm results in different geometric and electronic properties that strongly affect the adsorption and activation of the reactants, resulting in creation of more catalytic active sites with low-coordinated atoms that are usually located in the defects such as terraces, edges, kinks, and vacancies^36^.

**Figure 10:**
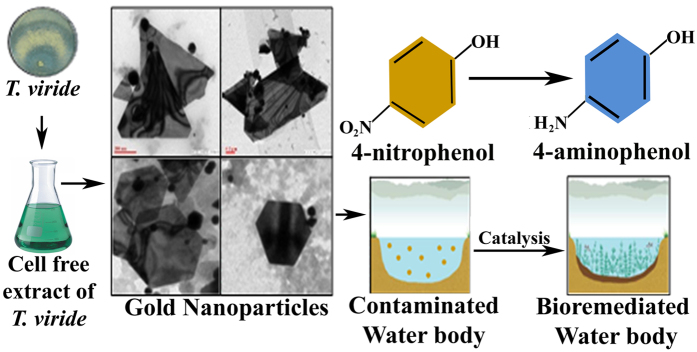
Overall graphical abstract illustrating diverse shapes biosynthesized GNP and its ability to degrade 4-nitropdhenol into 4-aminophenol.

The ability of heterogenous gold nanocatalysts to recycle themselves^39^ for further degradation of organic pollutants and enhanced selectivity and reactivity of biological nanocrystals than their conventional counterparts makes them a very promising candidate for application in biotransformation. Our work provides a detailed study of structure and dimension controlled biosynthesized GNP for the degradation of 4-nitrophenol into 4-aminophenol (Fig. 10).

## Conclusion

The cost-effective, simple and green protocols were developed to synthesize biogenic gold nanoparticles of different shapes and sizes with good yield and monodispersity. The peculiarity of our study is synthesis of gold nanoparticles of size 3–10 nm, having highest catalytic activity among the other different shapes and sizes of the particles by modulating the physical parameters. A complete control on physical and biological parameters (pH, time, temperature, concentration of salts and cell free extract) to obtained various shapes and sizes (spherical, penta/hexagons, triangle) were found during biosynthesis of GNPs. Deciphering of the unique balance of physical and biological factors networking to find highly reactive nanocatalyst, that bears a great potential in heterogenous catalysis may revolutionise the organic transformation for bioremediation.

## Materials and Methods

### Materials

The Gold (III) chloride (ACS reagent) was purchased from Sigma Aldrich, USA and used as received. All other reagents used were of analytical grade. All other reagents 4-Nitrophenol, 4- Aminophenol and NaBH_4_ were also from Sigma Aldrich, USA used were of analytical grade.

### Isolation of fungal isolate

*T. viride* (MTCC 5661) was isolated from CSIR-NBRI garden campus, and deposited in Microbial Type Culture Collection (MTCC), Chandigarh, India^16^. The culture was grown and maintained on Potato dextrose agar (PDA) medium at 28 °C for 4 days and maintained at 4 °C in refrigerator.

### Fungal cell free extract preparation and synthesis of gold nanoparticles

To prepare cell free extract, two bids of 6 mm were cut from the seven-day old culture of *T. viride* maintained on PDA and inoculated in 100 ml of potato dextrose broth (PDB). It was allowed to grow for4 days at 28 °C at 80 rpm. After 4 days, the biomass obtained was filtered through Whatman filter paper no. 1 and washed with autoclaved MQ water thrice. The biomass thus obtained was re-suspended in 100 ml of sterile deionised water for 3 days in similar conditions.The cell free extract was obtained by filtering out the biomass after 3 days.

### Morphological observations by varying biological and physical parameters

To evaluate the effect of various biological and physical parameters, the reaction was carried out under different reaction conditions.

#### Fungal cell free extract and gold salt preparation

Three different fungal cell free extract concentration (10, 50 and 100%) were used in this study. The cell free extract obtained without any dilution served as 100% concentrated cell free extract. For preparing 10 and 50% of concentration, 10 ml and 50 ml of 100% concentrated cell free extract were added to 90 ml and 50 ml of MQ in sterilized conditions. Two different concentration of HAuCl_4_ (250 and 500 mg/L) was prepared in sterilized MQ water for assessing the synthesis of gold nanoparticles.

#### Effect of pH

pH of the cell free extract was varied at 5.0, 7.0 and 9.0at different reaction temperature (20, 30, 40 and 50 °C) and different reaction time (24, 48 and 72 h) by addition of 0.1 M HCl or 0.1 M NaOH till the desired pH is reached. Further experiments were also conducted experiments were conducted in the pH range of 5.0 to 10.0 at an interval of 0.5 at 30 °C, 72 h.

#### Effect of reaction temperature and time

The reaction mixture obtained by altering various parameters like pH, substrate concentration and cell free extract, were kept in culture tubes, and incubated in rotary shaker at 150 rpm for different time intervals of 24, 48 and 72 h at different reaction temperature (20,30,40 and 50 °C). Samples were procured after specific time intervals for measurement of UV-vis spectrum, DLS and TEM size measurement.

### Characterization of gold nanoparticles

Preliminary characterization of GNPs synthesized was done by visual observation for change in colour of cell free extract, and further formation of GNPs was confirmed by UV-visible spectroscopy (Thermo spectrascan UV 2700) for appearance of characteristic surface plasmon resonance band of gold nanoparticles^40^. To study the hydrodynamic size and poly dispersity index (PDI) of the nanoparticles biosynthesized, particle size analyzer was used (Malvern, nanoseries zeta sizer, UK)^41^. Particle size and morphology were investigated using transmission electron microscopy (Technai G2 spirit, FEI, Netherland) with Gatan Orius camera. Samples were prepared by filtering them through 0.45 μ syringe millipore filters and sonicated for 2 min. After sonication, a drop of solution was placed immediately on formvar-coated copper grid and left overnight for drying. Bright field TEM studies were carried out at 80 KV. Size of ten different nanoparticles were measured randomly in one field during TEM studies and average size was calculated (n = 3). The crystalline phase was detected using X-ray diffraction (XRD) analysis. For fourier transform infra-red spectroscopy (FTIR) measurements, the nanoparticles were freeze dried and diluted with potassium bromide in the ratio of 1:100. The FTIR spectra of samples were recorded on a FTIR instrument (Agilent Cary 630, USA). All measurements were carried out in the range of 400–4,000 cm^−1^.

### Effect of different morphologies of GNP in catalytic degradation of 4-nitrophenol

Effect of different shapes and sizes of biosynthesized GNPs on their catalytic activity to reduce 4NP into 4AP was assessed by the method of Gangula *et al.*^42^, with some minor modifications. In a 3 ml of quartz cuvette, 1.7 mL of water, 0.3 mL of 2 mM solution of 4-nitrophenol and 1 ml of 0.03 M of freshly prepared NaBH_4_ solution were added^43^,^44^. To this reaction mixture, 50 μL of gold nanoparticles of different morphologies viz. spherical, triangular, hexagonal and sheet shaped were added. The reaction temperature was kept constant at room temperature (25 °C) to avoid the thermal effect on the process of catalysis. The reaction mixture was stirred well with microstirrer and quickly scanned between 200–600 nm in UV-vis spectroscopy (Thermo spectrascan UV 2700). Further, to assess the effect of size, spherical gold nanoparticles of two different size range (3–10 and 7–24 nm) were used as biocatalyst. The 4-nitrophenol shows an absorbance peak at 400 nm in presence of NaBH_4_ due to formation of nitrophenolate ions, so the progress of the reaction was monitored by tracking the decrease in the absorption spectra of 4-nitrophenolate ion at 400 nm. Kinetics of the reaction was monitored for 15 min which showed a decrease in absorbance at 400 nm and increase in absorbance at 290 nm at an interval of 1 nm.

### Gas chromatography- Mass spectrometric (GC) analysis for catalytic conversion of p-nitrophenol into p-aminophenol

Catalytic degradation of 4-nitrophenol into 4-aminophenol by gold nanoparticles (Spherical gold nanoparticles showing highest reaction kinetics; 3–10 nm and 7–20 nm) were confirmed by gas chromatography mass-spectrometric analysis (Trace GC Ultra TSQ Quantum XLS, Thermo Scientific). Reaction mixtures (4-NP + GNP + NABH_4_) were harvested at time intervals of 0, 5, 10, 20, 30 min. then the reaction was stopped by using 2 M HCl and the resultant mixture was, lyophilized (Thermo Scientific Heto Freeze Dryer 1.0–110) and subjected to GC-MS/MS analysis. The instrument was equipped with Tri-Plus auto-sampler to perform injector port silylation at the injector port of GC. The instrument was equipped with DB-5 MS capillary column, and ionization voltage was 70 eV. An aliquot of 1.5 μl of sample was injected and analysed using the following condition. The oven temperature programming was set initially at 100 °C for 1 min, then increased to 160 °C at the rate of 20 °C/min after that increased to 175 °Cwith a rate of 2 °C/min, and then the final temperature was set at up to with a 290 °C with a rate of 60 °C/min. The analysis was done in the full scan mode of GC-MS and the confirmation of peaks done by using Selected Reaction Mode (SRM) at a transitions of 253→238 for 4-AP and 211→196 for 4-NP with a collision energy of 15. The calibration curve was constructed for 4-AP and 4-NP in the concentration of range of 0.01–10 μg/mL and found to be linear with a regression coefficient of 0.994 and 0.998 respectively (Fig. S17).

## Additional Information

**How to cite this article**: Kumari, M. *et al.* Physico-Chemical Condition Optimization during Biosynthesis lead to development of Improved and Catalytically Efficient Gold Nano Particles. *Sci. Rep.* **6**, 27575; doi: 10.1038/srep27575 (2016).

## Supplementary Material

Supplementary Information

## Figures and Tables

**Table 1:** Effect of different physico-chemical parameters on biosynthesis of gold nanoparticles.

Sample code	Physico-Chemical Condition Optimization						
	Cell free extract concentration (%)	Conc. of gold salt (mg/L)	pH	Time(h)	Temp( °C)	TEM size(nm)	Shape
Effect of cell free extract concentration (%)							
G10.250.7.72.30	10	250	7	72	30	34 ± 20	Mixed
G50.250.7.72.30	50	250	7	72	30	104 ± 55	Mixed
G100.250.7.72.30	100	250	7	72	30	197.58 ± 68	Mixed
Effect of concentration of gold salt (mg/L) and Temp(°C)							
G10.250.7.72.30	10	250	7	72	30	34 ± 20	Mixed
G10.500.7.72.30	10	500	7	72	30	84.77 ± 2.38	Hexagons
G10.250.7.72.40	10	250	7	72	40	164.90 ± 96.38	Triangles
G10.500.7.72.40	10	500	7	72	40	337.41 ± 163.09	Triangles
G10.250.7.72.50	10	250	7	72	50	277.0595 ± 163.18	Triangles
G10.500.7.72.50	10	500	7	72	50	671.8925 ± 246.95	Triangles
Effect of pH, gold salt concentration and Temp( °C)							
G10.250.5.72.20	10	250	5	72	20	92.7 ± 47.83	Mixed
G10.250.5.72.30	10	250	5	72	30	61.08 ± 60.00	Mixed
G10.250.5.72.40	10	250	5	72	40	43 ± 12.37	Pentagons and triangles
G10.250.5.72.50	10	250	5	72	50	255.65 ± 78.56	Triangles
G10.500.5.72.20	10	500	5	72	20	42.5 ± 40.5	Mixed
G10.500.5.72.30	10	500	5	72	30	25.5636 ± 7.48	Pseudospheres
G10.500.5.72.40	10	500	5	72	40	91.65 ± 62.51	Pentagons and triangles
G10.500.5.72.50	10	500	5	72	50	475.5 ± 242.40	Triangles
G10.250.7.72.20	10	250	7	72	20	10.25 ± 2.1	Sheets
G10.250.7.72.30	10	250	7	72	30	34 ± 20	Mixed
G10.250.7.72.40	10	250	7	72	40	69.65 ± 58.72	Triangles
G10.250.7.72.50	10	250	7	72	50	215 ± 89.55	Triangles
G10.500.7.72.20	10	500	7	72	20	36 ± 5.09	Pseudospheres
G10.500.7.72.30	10	500	7	72	30	84.77 ± 2.38	Hexagons
G10.500.7.72.40	10	500	7	72	40	337.41 ± 163.09	Triangles
G10.500.7.72.50	10	500	7	72	50	671.8925 ± 246.95	Triangles
G10.250.9.72.20	10	250	9	72	20	32.5 ± 12.68	Mixed
G10.250.9.72.30	10	250	9	72	30	6.425 ± 2.1	Spheres
G10.250.9.72.40	10	250	9	72	40	88.5 ± 17.56	Mixed
G10.250.9.72.50	10	250	9	72	50	356.5 ± 123.59	Triangles
G10.500.9.72.20	10	500	9	72	20	15 ± 6.88	Mixed
G10.500.9.72.30	10	500	9	72	30	19 ± 4.08	Spheres
G10.500.9.72.40	10	500	9	72	40	84.15 ± 14.70	Penta and hexagons
G10.500.9.72.50	10	500	9	72	50	529.15 ± 262.46	Triangles
Effect of pH							
G10.250.5.72.30	10	250	5	72	30	61.08 ± 60.00	Mixed
G10.250.5.5.72.30	10	250	5.5	72	30	45.68 ± 23.46	star/hexagonal
G10.250.6.72.30	10	250	6	72	30	47 ± 28.64	Triangles
G10.250.6.5.72.30	10	250	6.5	72	30	84.2 ± 61.20	Triangles
G10.250.7.72.30	10	250	7	72	30	34 ± 20	Mixed
G10.250.7.5.72.30	10	250	7.5	72	30	46.65 ± 34.84	Triangles
G10.250.8.72.30	10	250	8	72	30	41.5855 ± 12.27	Triangles
G10.250.8.5.72.30	10	250	8.5	72	30	59.4374 ± 11.67	penta/hexagons
G10.250.9.72.30	10	250	9	72	30	6.425 ± 2.1	Spheres
G10.250.9.5.72.30	10	250	9.5	72	30	4 ± 1.74	Irregular
G10.250.10.72.30	10	250	10	72	30	4.6 ± 0.98	Irregular
Effect of pH and Time(h)							
G10.250.5.24.30	10	250	5	24	30	11.753 ± 4.26	Pseudospheres
G10.250.5.48.30	10	250	5	48	30	40.53 ± 39.03	Mixed
G10.250.5.72.30	10	250	5	72	30	61.08 ± 60.00	Mixed
G10.250.7.24.30	10	250	7	24	30	57.345 ± 24.93	Mixed
G10.250.7.48.30	10	250	7	48	30	27 ± 25.01	Mixed
G10.250.7.72.30	10	250	7	72	30	34 ± 20	Mixed
G10.250.9.24.30	10	250	9	24	30	5 ± 0.79	Spheres
G10.250.9.48.30	10	250	9	48	30	5 ± 1.24	Spheres
G10.250.9.72.30	10	250	9	72	30	6.425 ± 2.1	Spheres

Sample code denotes the physico chemical parameters for synthesis of gold nanoparticles. For example in sample code G10.250.7.72.30, G denotes gold nanoparticles, 10 denotes percent of cell free extract, 250 denotes concentration of gold salt in mg/L, 7 denotes pH, 72 denotes reaction time in hour, and 30 denotes temperature in °C.
