# Supplementary material for: Physico-Chemical Condition Optimization during Biosynthesis lead to development of Improved and Catalytically Efficient Gold Nano Particles
Source: Sci Rep. 2016 Jun 8;6:27575. doi: 10.1038/srep27575 (PMC4897682; doi:10.1038/srep27575)
Supplement: Supplementary Information [file srep27575-s1.pdf]

Supplementary Information

Physico-Chemical Condition Optimization during Biosynthesis lead to development of Improved and Catalytically Efficient Gold Nano Particles

Madhuree Kumari[1], Aradhana Mishra[1], Shipra Pandey[1], Satyendra Pratap Singh[1], Vasvi Chaudhry[1], Mohana Krishna Reddy Mudiam[2], Shatrunajay Shukla[2], Poonam Kakkar[2], C.S. Nautiyal[1]*

[1]CSIR-National Botanical Research Institute, Rana Pratap Marg, Lucknow, 226 001, India

[2]CSIR-Indian Institute of Toxicology Research, 80, Mahatma Gandhi Marg, Lucknow, 226 001, India

*Corresponding author:

Dr C. S. Nautiyal

Rana Pratap Marg, Lucknow, 226 001, India.

E mail: nautiyalnbri@lycos.com

Tel/ Fax: 91 522 2206651

Supplementary Figures:

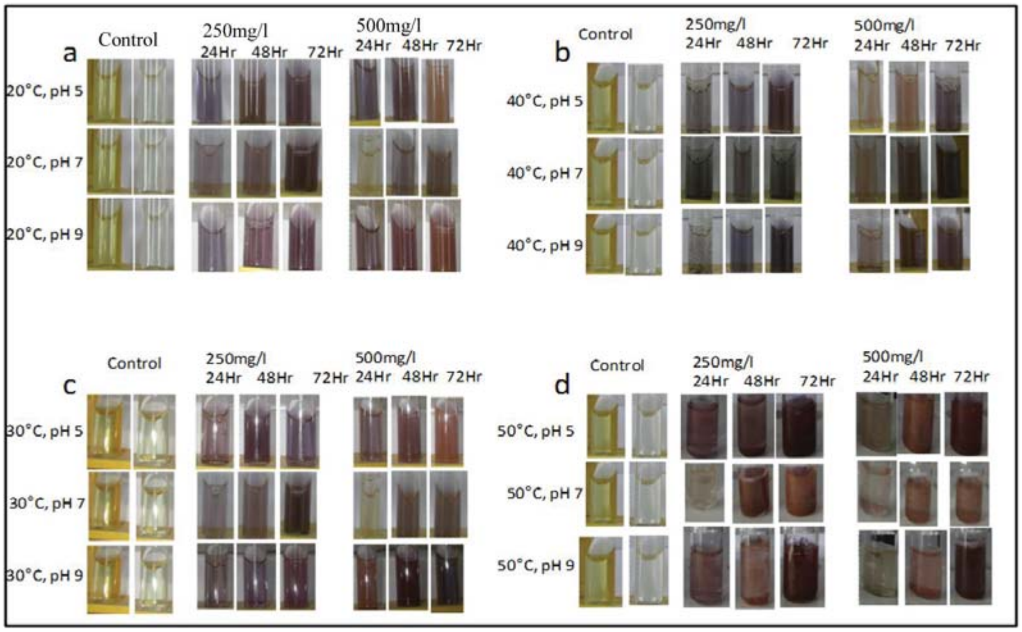

Figure S1. Color image of gold nanoparticles biosynthesized at different reaction conditions, pH 5.0, 7.0 and 9.0, time 24, 48 and 72h, $HAuCl_4$ concentration- 250 and 500 mg/L **(a)** at 20°C, **(b)** at 30°C, **(c)** at 40°C, **(d)** at 50°C.

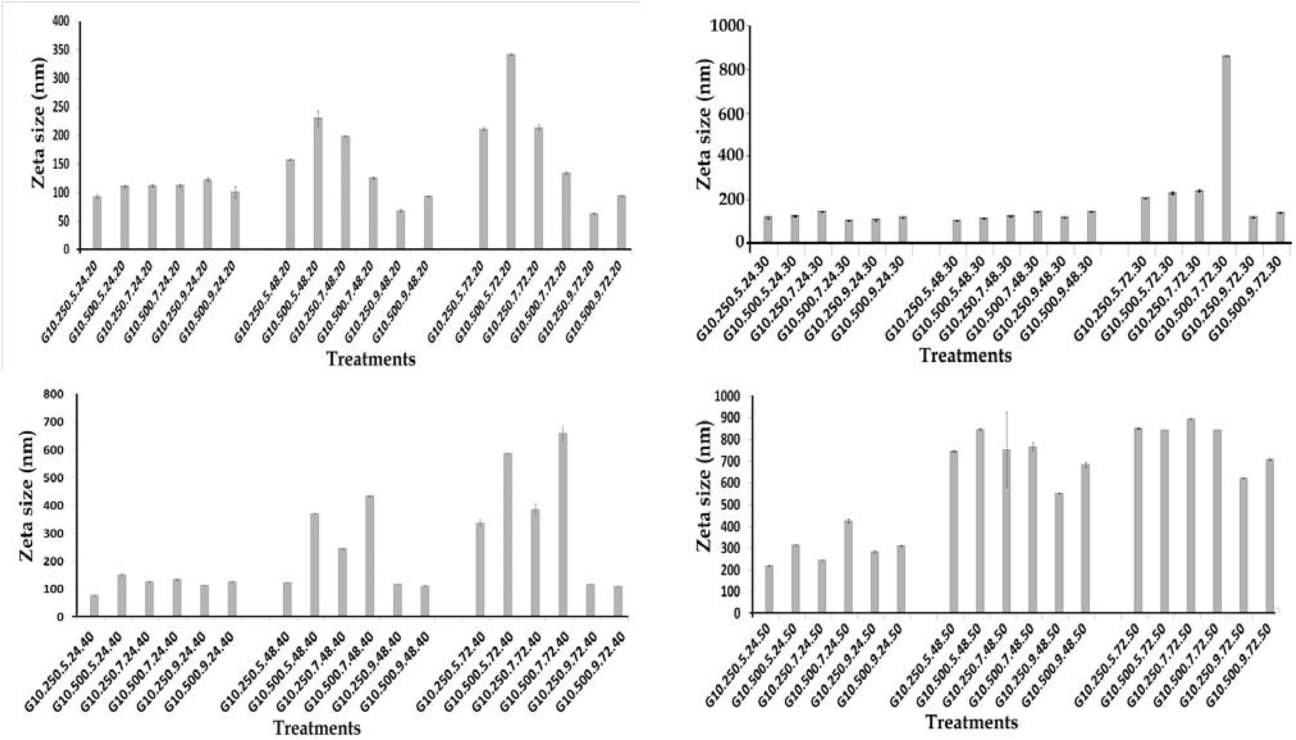

Figure S2. Zeta size of gold nanoparticles biosynthesized at different reaction conditions, pH 5.0, 7.0 and 9.0, time 24, 48 and 72h, $HAuCl_4$ concentration- 250 and 500 mg/L **(a)** at 20 °C, **(b)** at 30 °C, **(c)** at 40 °C, **(d)** at 50 °C.

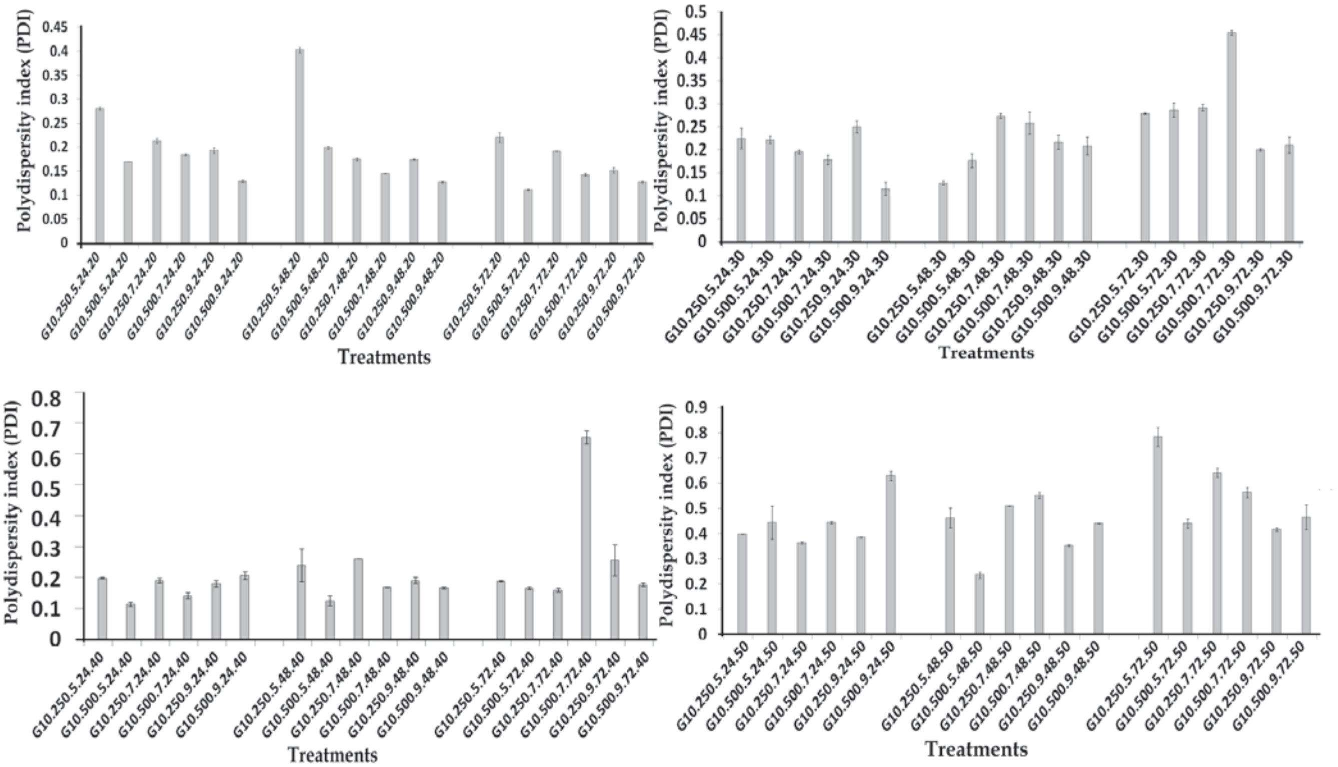

Figure S3. Poly dispersity index (PDI) of gold nanoparticles biosynthesized at different reaction conditions, pH 5.0, 7.0 and 9.0, time 24, 48 and 72h, $HAuCl_4$ concentration- 250 and 500 mg/L **(a)** at 20°C, **(b)** at 30°C, **(c)** at 40°C, **(d)** at 50°C.

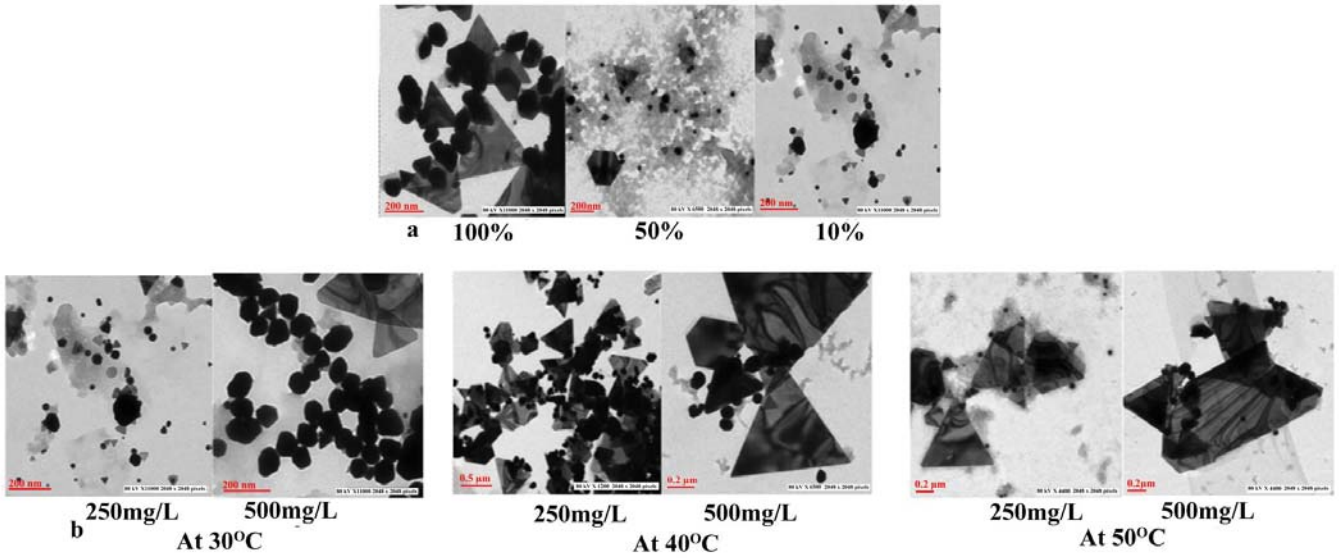

Figure S4. TEM micrographs of **(a)** G100.250.7.72.30, G50.250.7.72.30 and G10.250.7.72.30 showing effect of different concentration of cell free extracts and **(b)** G10.250.7.72.30, G10.500.7.72.30, G10.250.7.72.40, G10.500.7.72.40, G10.250.7.72.50 and G10.500.7.72.50 showing effect of $HAuCl_4$ (250 and 500 mg/L) at different reaction temperatures on the shape and size of gold nanoparticles.

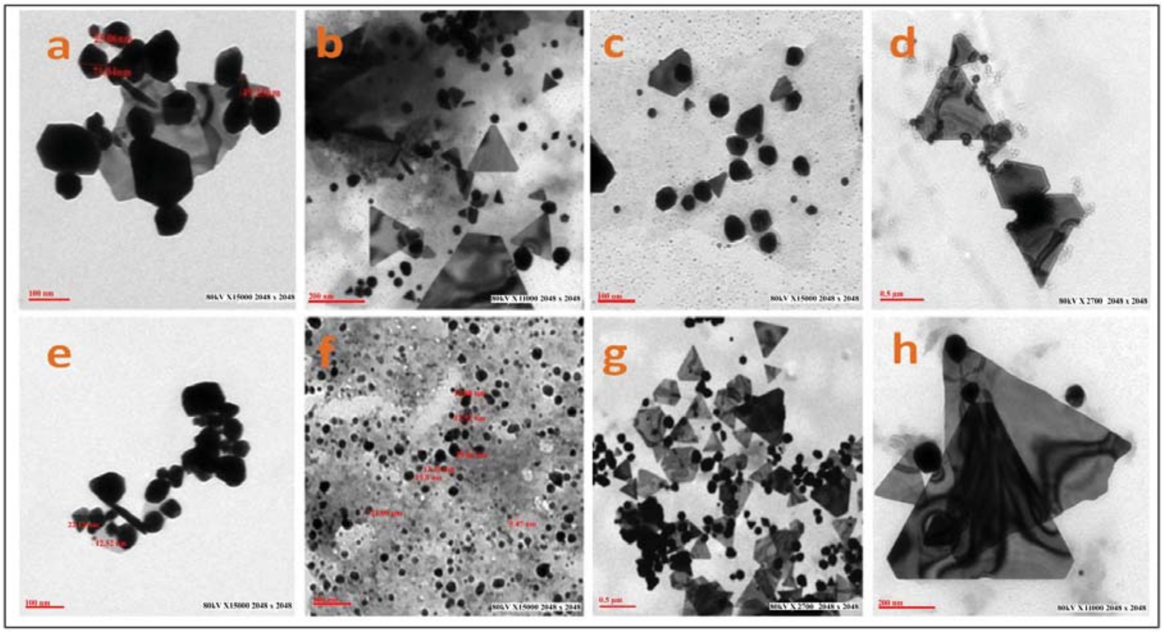

Figure S5. TEM micrographs of (a) G10.250.5.72.20 (b) G10.250.5.72.30 (c) G10.250.5.72.40 (d) G10.250.5.72.50 (e) G10.500.5.72.20 (f) G10.500.5.72.30 (g) G10.500.5.72.40 (h) G10.500.5.72.50 showing effect of different reaction temperatures at pH 5.0, at different concentration of $HAuCl_4$ on shape and size of gold nanoparticles (See table 1 for detail).

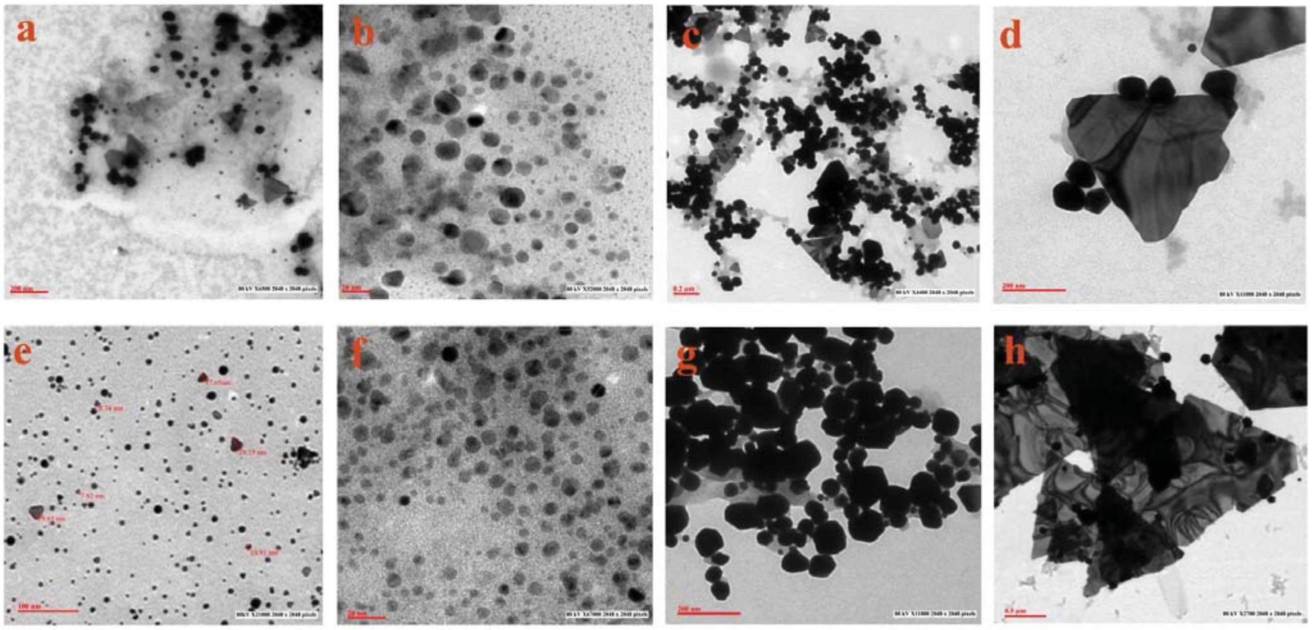

Figure S6. TEM micrographs of (a) G10.250.9.72.20 (b) G10.250.9.72.30 (c) G10.250.9.72.40 (d) G10.250.9.72.50 (e) G10.500.9.72.20 (f) G10.500.9.72.30 (g) G10.500.9.72.40 (h) G10.500.9.72.50 showing effect of different reaction temperatures at pH 9.0, at different concentration of HAuCl4 on shape and size of gold nanoparticles (See table 1 for detail).

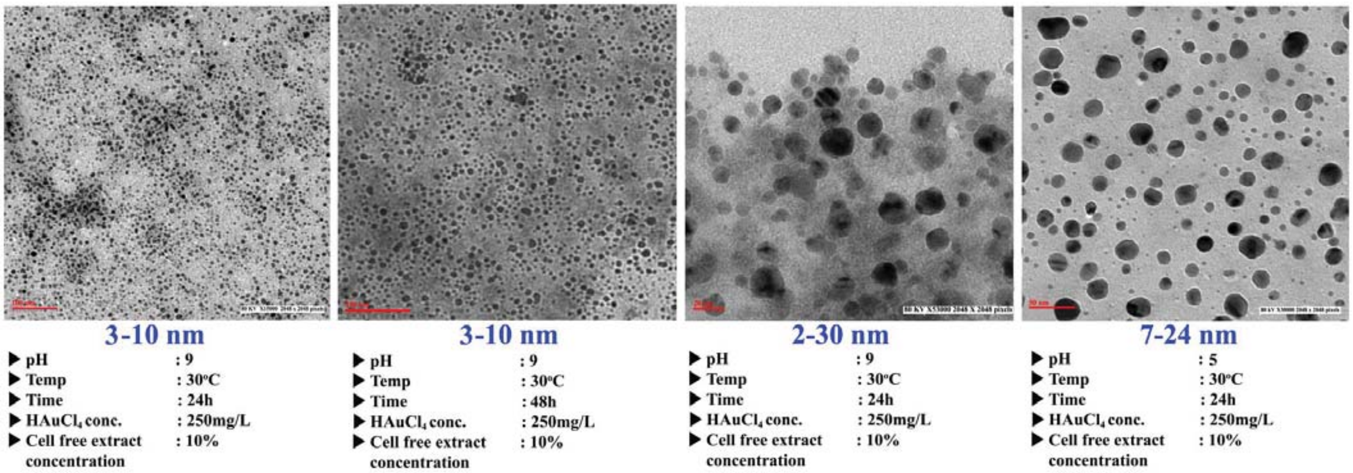

Figure S7. TEM micrographs and their respective biological and physical conditions for biosynthesis of spherical gold nanoparticles of smallest size.

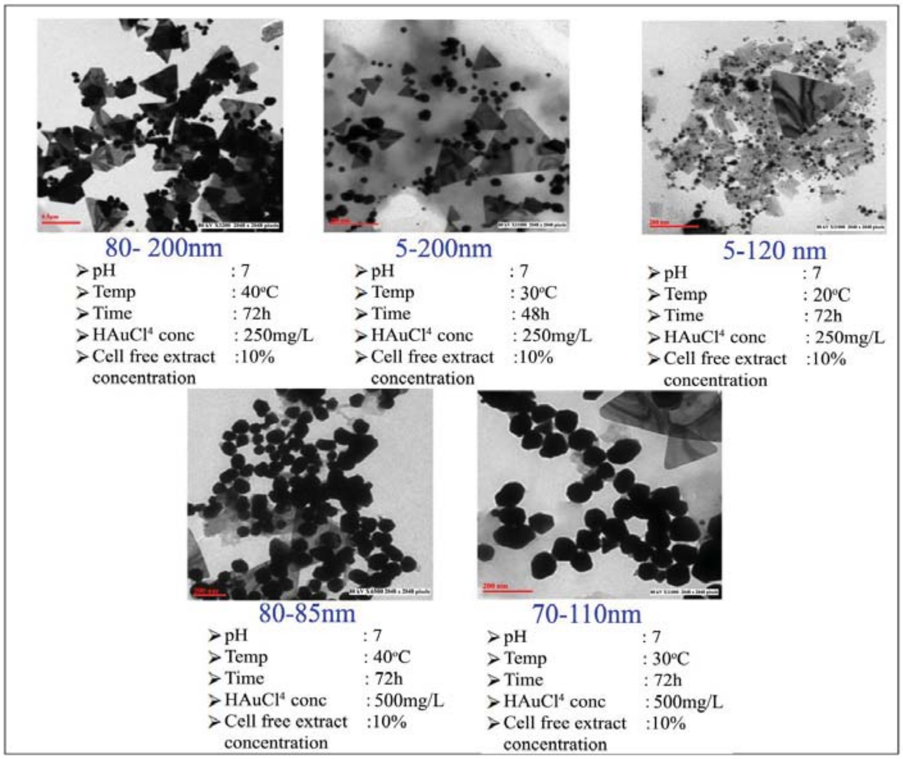

Figure S8. TEM micrographs and their respective biological and physical conditions for biosynthesis of gold nanoparticles of various shapes.

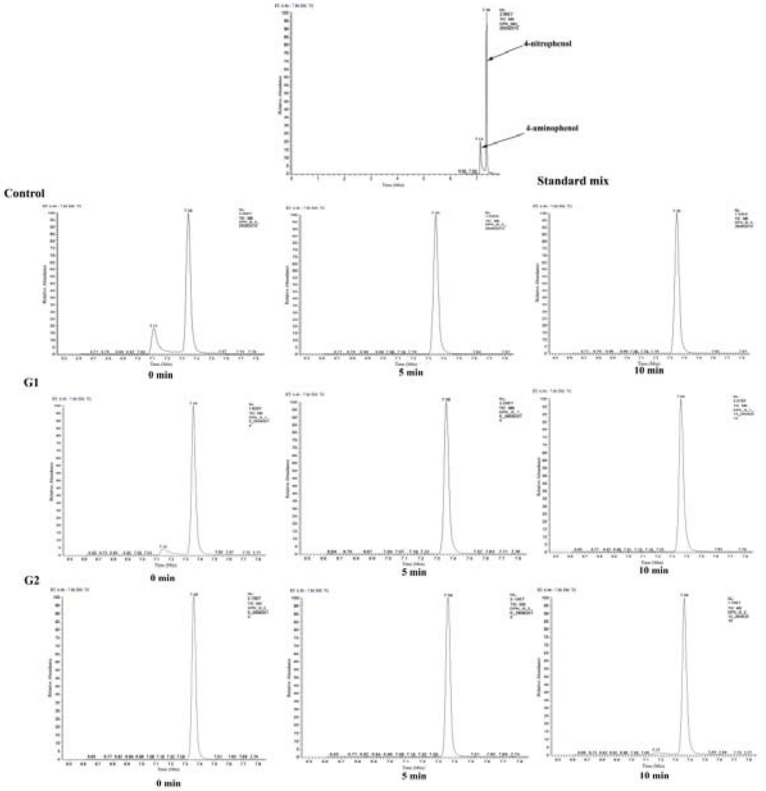

Figure S9. GC-MS/MS analysis of degradation of 4-nitrophenol into 4-aminophenol by different sphere sizes of biosynthesized GNP.

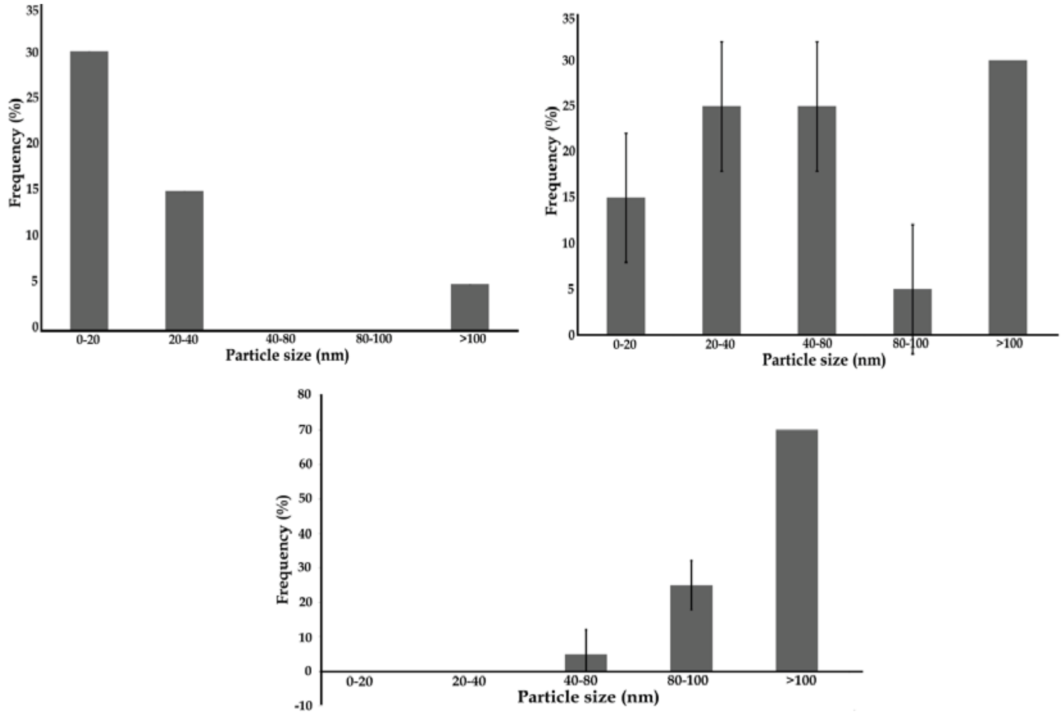

Figure S10. Frequency distribution graph of G100.250.7.72.30, G50.250.7.72.30 and G10.250.7.72.30 showing effect of different concentration of cell free extracts (100, 50 and 10%)

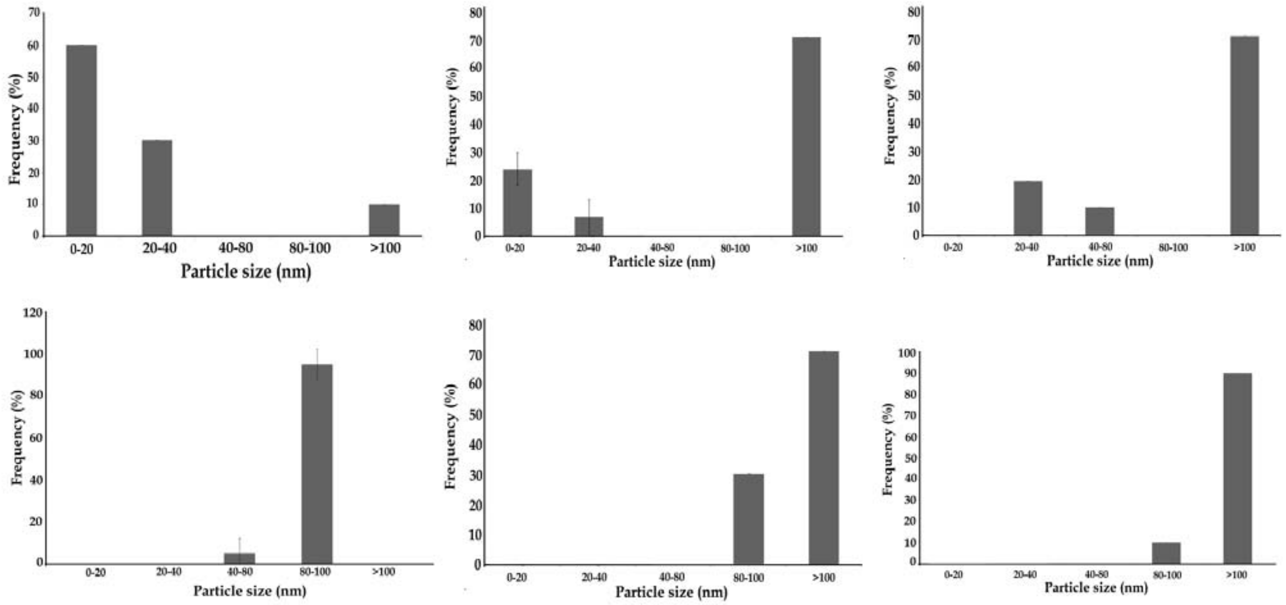

Figure S11. Frequency distribution graph of (a) G10.250.7.72.30 (b) G10.250.7.72.40 (c) G10.250.7.72.50 (d) G10.500.7.72.30 (e) G10.500.7.72.40 (f) G10.500.7.72.50 showing effect of different gold concentration on shapes and sizes of gold nanoparticles

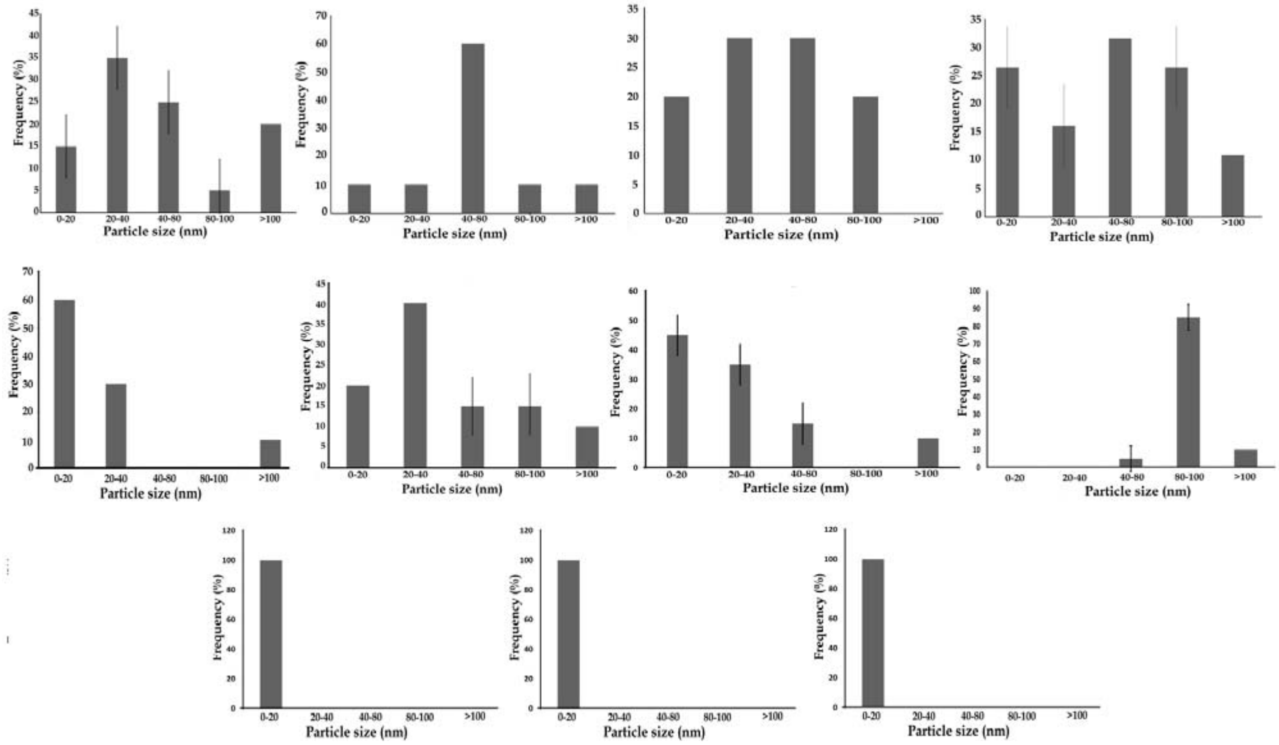

Figure S12. Frequency distribution graph of (a) G10.250.5.72.30 (b) G10.250.5.5.72.30 (c) G10.250.6.72.30 (d) G10.250.6.5.72.30 (e) G10.250.7.72.30 (f) G10.250.7.5.72.30 (g) G10.250.8.72.30 (h) G10.250.8.5.72.30 (i) G10.250.9.72.30 (j) G10.250.9.5.72.30 (k) G10.250.10.72.30 showing effect of different pH on shapes and sizes of gold nanoparticles (See table 1 for detail).

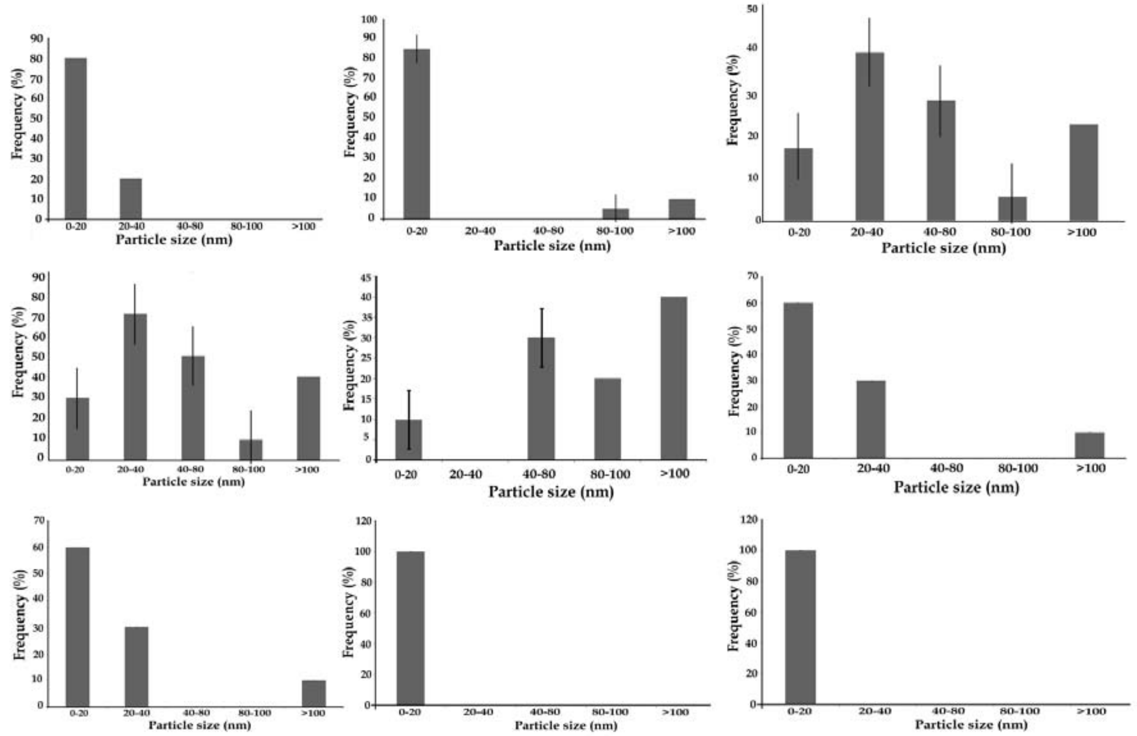

Figure S13. Frequency distribution graph of (a) G10.250.5.24.30 (b) G10.250.5.48.30 (c) G10.250.5.72.30 (d) G10.250.7.24.30 (e) G10.250.7.48.30 (f) G10.250.7.72.30 (g) G10.250.9.24.30 (h) G10.250.9.48.30 (i) G10.250.9.72.30 showing effect of different time intervals at different pH shapes and sizes of gold nanoparticles (See table 1 for detail).

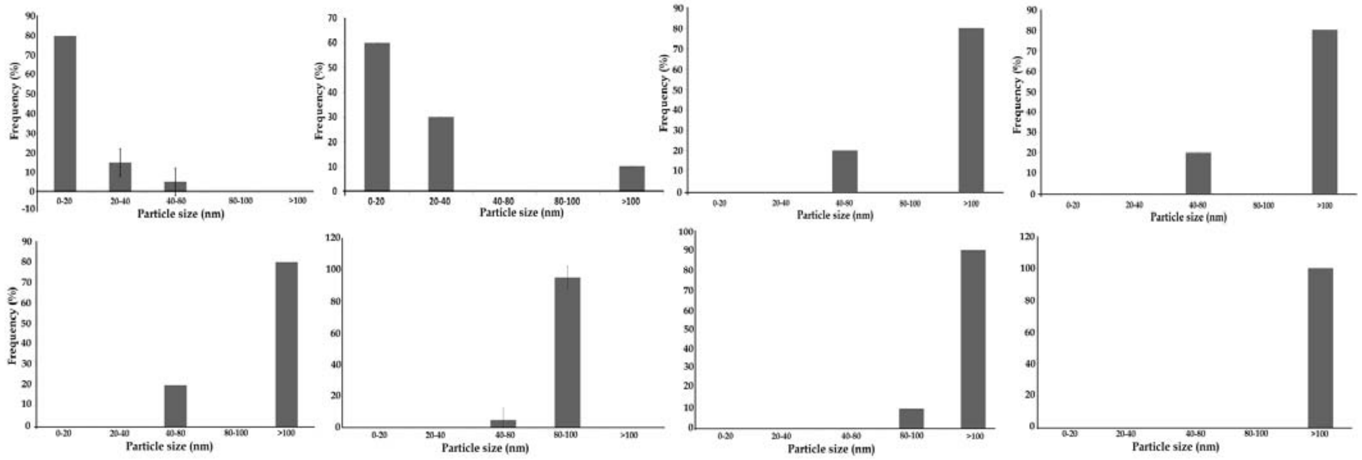

Figure S14. Frequency distribution graph of (a) G10.250.7.72.20 (b) G10.250.7.72.30 (c) G10.250.7.72.40 (d) G10.250.7.72.50 (e) G10.500.7.72.20 (f) G10.500.7.72.30 (g) G10.500.7.72.40 (h) G10.500.7.72.50 showing effect of different reaction temperatures at pH 7.0, at different concentration of $HAuCl_4$ on shape and size of gold nanoparticles (See table 1 for detail).

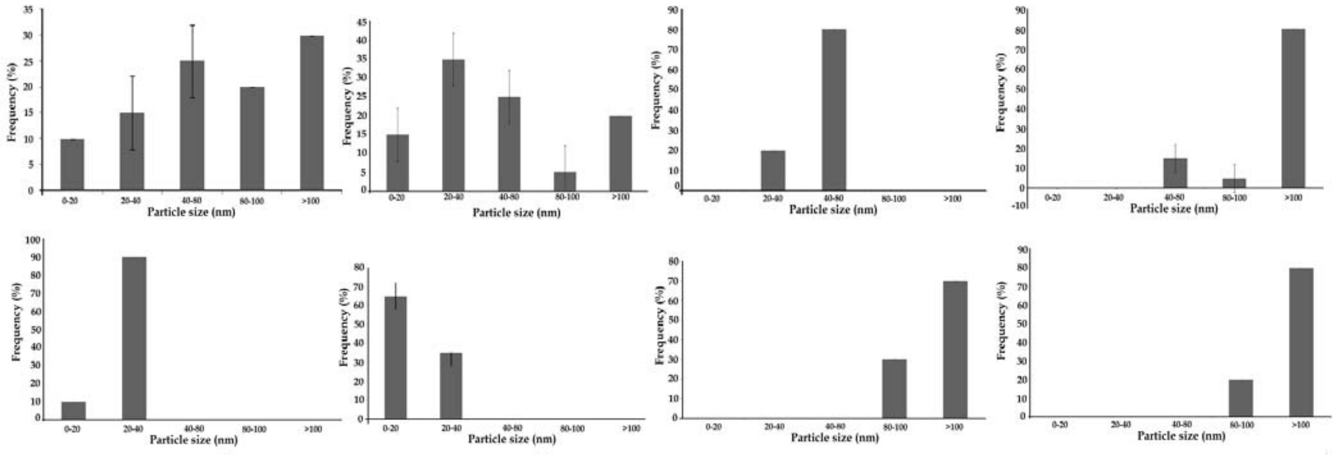

Figure S15. Frequency distribution graph of (a) G10.250.5.72.20 (b) G10.250.5.72.30 (c) G10.250.5.72.40 (d) G10.250.5.72.50 (e) G10.500.5.72.20 (f) G10.500.5.72.30 (g) G10.500.5.72.40 (h) G10.500.5.72.50 showing effect of different reaction temperatures at pH 5.0, at different concentration of HAuCl4 on shape and size of gold nanoparticles (See table 1 for detail).

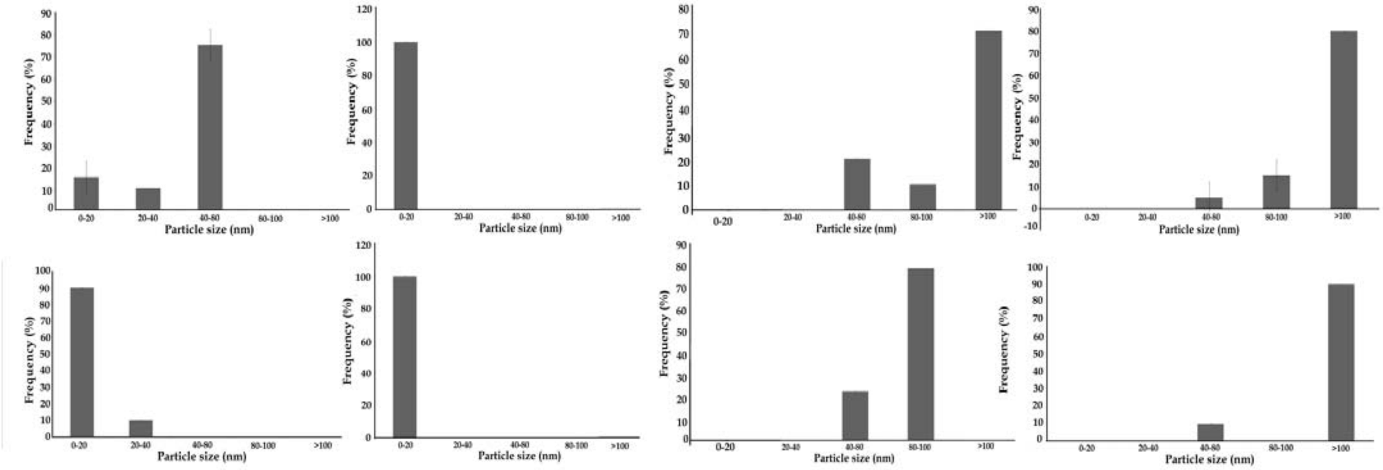

Figure S16. Frequency distribution graph of (a) G10.250.9.72.20 (b) G10.250.9.72.30 (c) G10.250.9.72.40 (d) G10.250.9.72.50 (e) G10.500.9.72.20 (f) G10.500.9.72.30 (g) G10.500.9.72.40 (h) G10.500.9.72.50 showing effect of different reaction temperatures at pH 9.0, at different concentration of $HAuCl_4$ on shape and size of gold nanoparticles (See table 1 for detail).

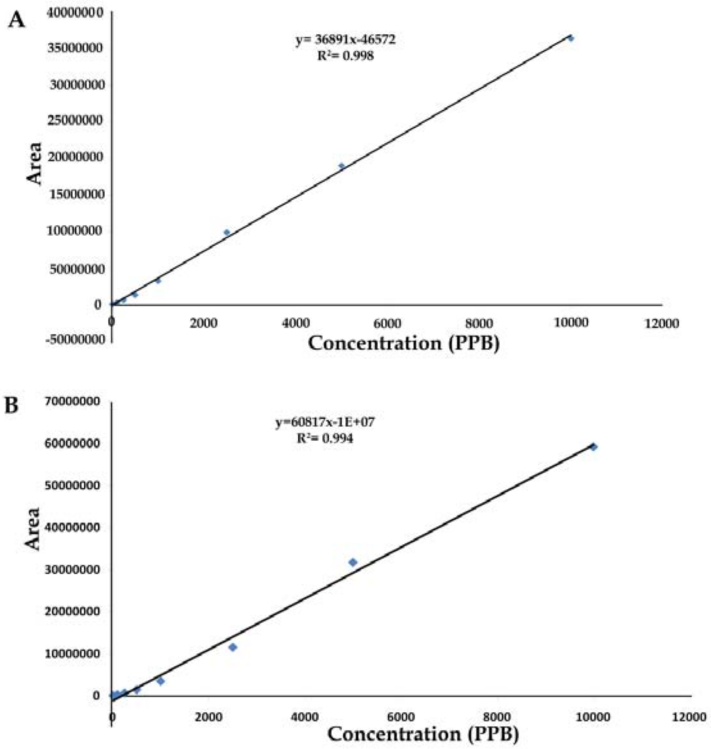

Figure S17. The calibration graph of (A) 4-NP (B) 4-AP obtained by GC-MS study.
